# Uric acid and evaluate the coronary vascular stenosis gensini score correlation research and in gender differences

**DOI:** 10.1186/s12872-023-03581-5

**Published:** 2023-11-08

**Authors:** Bao Yang, Kanghua Ma, Rui Xiang, Guoli Yang, Yue Luo, Fan Wu, Min Mao

**Affiliations:** Department of Cardiology, The First Affiliated Hospital of Chongqing Medical University, Yuzhong, Chongqing, 400010 China

**Keywords:** Coronary artery Disease, Gensini score, Serum uric acid, Coronary arteriography

## Abstract

**Background and aims:**

Recent studies have shown that the negative effect of uric acid (UA) on coronary arteries determines the severity of atherosclerotic disease. This study aims to explore the relationship between serum UA level and Gensini score, which reflects the severity of coronary artery disease.

**Methods:**

A total of 860 patients with suspected coronary heart disease who were admitted to hospital due to angina pectoris or myocardial ischemia related symptoms and received coronary angiography were selected. Based on the findings of the angiography, they were categorized into two groups: the coronary heart disease (CHD) group (n = 625) and the control group (n = 235). The uric acid levels and other clinical data were compared between these groups. Additionally, the prevalence of coronary heart disease and Gensini score were compared between the groups, considering gender-specific quartiles of uric acid levels. The clinical baseline data were analyzed using appropriate statistical methods, and multivariate logistic regression analysis was conducted to identify independent risk factors for coronary heart disease.

**Results:**

Of 860 patients (mean age, 63.97 ± 11.87 years), 528 were men (mean age, 62.06 ± 11.5 years) and 332 were women (mean age, 66.99 ± 10.11 years). The proportion of smoking, diabetes, hypertension, and hyperlipidemia in the coronary heart disease group was higher than that in the control group (*P* < 0.05). HbA1C, Gensini score, BMI, TG and hsCRP in the coronary heart disease group were higher than those in the control group (*P* < 0.05), and HDL-C was lower than that in the control group (*P* < 0.05). There were no significant differences in age, heart rate, Cr, TC and LDL-C between the two groups *(P* > 0.05).Multivariate logistic regression analysis showed that age, hypertension, hsCRP and SUA levels increased the risk of coronary heart disease, and the difference was statistically significant(OR = 1.034,95%CI 1.016–1.052, *P* = 0.001; OR = 1.469,95%CI 1.007–2.142, *P* = 0.046;OR = 1.064,95%CI 1.026–1.105, *P* = 0.001; OR = 1.011,95%CI 1.008–1.014, *P* < 0.001).

**Conclusion:**

Serum uric acid is positively correlated with Gensini score in patients with coronary heart disease, which is an independent factor for evaluating the degree of coronary artery stenosis and has a predictive effect.

## Introduction

Coronary heart disease is a condition characterized by atherosclerosis, resulting in significant stenosis (≥ 50%) of major coronary arteries such as the left main artery, left anterior descending artery, left circumflex artery, and right coronary artery. This stenosis leads to myocardial ischemia and hypoxia, profoundly impacting both the structure and function of the myocardium, eventually triggering the onset of the disease. Following an episode, it manifests as angina, dyspnea, and chest tightness. In severe instances, it may be accompanied by heart failure, posing a threat to one’s life safety [1, 2]. Hence, coronary artery disease poses a significant public health menace in China and presents an escalating global health burden, thus being a major contributor to mortality rates [3–7]. Currently, over 200 risk factors associated with coronary heart disease have been identified. The risk factors for cardiovascular disease initially introduced by the Framingham Heart Study in 1961 are referred to as conventional risk factors, encompassing aspects such as age, tobacco use, excessive weight, elevated blood pressure, high blood sugar levels, abnormal lipid levels, and sedentary lifestyle [8–11]. Ever since that time, numerous cardiovascular risk factors have emerged as novel (or non-conventional) risk factors, pertaining to the distinctive biomarkers of inflammation, oxidative stress, and insulin resistance. These factors include but are not limited to metabolic syndrome, sleep apnea, abnormalities in the blood coagulation and fibrinolysis systems, depression, anxiety, individuals with a low socioeconomic status, insufficiency of trace elements, hyperhomocysteinemia, and hyperuricemia [11, 12].

SUA serves as the ultimate outcome of purine metabolism within the human body. The elevation in SUA concentration primarily arises from the perturbation of the dynamic equilibrium between the synthesis and elimination of uric acid [13]. In recent times, driven by China’s burgeoning economy, the shifting dynamics of individuals’ lifestyles and dietary preferences, the incidence of hyperuricemia (HUA) has witnessed a marked increase [14]. SUA interacts with many other metabolic factors such as obesity, hypertension, glucose metabolism disorders and lipid metabolism disorders [15, 16].

The association of uric acid with gout and kidney disease has long been known, and new data suggest that uric acid is also associated with cardiovascular disease. In fact, UA is recognized as an important determinant of many different outcomes in the cardiovascular field, such as all-cause and CV mortality, acute coronary syndrome, chronic coronary syndrome, and stroke. In addition, UA is associated with the development of heart failure (HF), and in this group of patients is associated with higher mortality as well as the development of atrial fibrillation [17–19]. Although experimental studies have demonstrated that elevated uric acid levels result in impairment of vascular endothelial function through various mechanisms, including heightened inflammation and oxidative stress, eventually leading to the development of coronary heart disease, the clinical correlation between serum uric acid (SUA) levels and CHD might be undermined by numerous confounding variables. Consequently, the debate persists regarding the role of SUA as a diagnostic marker, a risk factor, or an active participant in the pathogenesis of CHD, and its potential utility in the diagnosis of this condition remains uncertain [20].

It has been observed that hypertension, hyperglycemia, obesity, smoking, and hyperuricemia play a role in the development of coronary heart disease, although the underlying mechanisms remain unclear in certain cases. There is a rising prevalence of coronary heart disease, with a noticeable trend towards affecting younger individuals [21]. Given the inconsistency in defining the severity of coronary artery disease across various studies, as well as the lack of correlation analysis between serum uric acid (SUA) levels and the severity of coronary artery disease in individuals with coronary heart disease in previous research, the relationship between SUA and the severity of coronary artery disease remains controversial. Currently, both domestic and international scholars have directed their attention to the relationship between SUA and coronary heart disease in patients who have undergone coronary angiography. This research usually involves dividing subjects into a coronary heart disease group and a control group, comparing their SUA levels. However, whether SUA levels have an impact on the extent of coronary artery stenosis among patients with coronary heart disease of different genders is still unclear. In this study, the Gensini score, which assesses the degree of coronary artery stenosis, was employed to evaluate the severity of vascular stenosis. The aim is to investigate the association between uric acid levels and coronary artery stenosis in patients with suspected coronary heart disease (CHD), and to further explore gender-related differences in these patients. Ultimately, this study intends to provide clinical evidence for early prediction, diagnosis, and treatment of CHD.

## Materials and methods

### Study participants

A total of 860 patients who underwent coronary angiography due to suspected coronary heart disease in the Cardiac Catheterization Laboratory of the First Affiliated Hospital of Chongqing Medical University from January 2021 to May 2022 were selected as the research subjects. Inclusion criteria: Coronary angiography was performed in patients with suspected coronary artery disease. Coronary artery disease was defined as at least one coronary artery with stenosis of ≥ 50% on coronary angiography. Suspected coronary heart disease refers to patients with angina or myocardial ischemia related symptoms, and there is some possibility of coronary artery disease. Specifically, the following may be considered to indicate suspected coronary heart disease: Classic angina: persistent or paroxysmal pressuring pain in the chest, usually radiating into the left shoulder, back, or jaw, after exertion or emotion. Atypical angina pectoris: Chest pain does not meet the characteristics of typical angina pectoris, but there are symptoms related to myocardial ischemia, such as chest discomfort, chest tightness, less severe chest pain, and short duration of pain. Resting angina pectoris: the patient has chest pain or angina in a quiet state, such as pain at night, pain when awake in the morning, etc. These patients were indicated for coronary angiography to assess the extent and lesion of coronary artery disease (coronary artery disease). Clinical data and biochemical indicators were collected by questionnaire survey through the electronic medical record system. Exclusion criteria: Previous diagnosis of hyperuricemia or gout; Taking urate-lowering medications; Kidney failure or other severe kidney disease, i.e. GFR < 30ml/min; Patients with fever, cold and other symptoms within 2 weeks or taking antibiotics; NYHA class III and above heart failure; Patients with secondary diseases causing elevated serum uric acid, such as blood diseases with hypermetabolism of nucleic acid, thyroid diseases, diabetes insipidus and after radiotherapy and chemotherapy of various malignant tumors; Cancer and so on. The Ethical Review Board of the First Affiliated Hospital of Chongqing Medical University approved the study protocol.

### Data collection

General information collection: a questionnaire was used to obtain the general demographic characteristics of the enrolled patients, including name, gender, age, living habits (such as smoking history, drinking history, etc.), medication, etc. Blood pressure, heart rate, height and weight were measured, and body mass index (BMI) was calculated. Coronary heart disease, hyperuricemia, hypertension, diabetes mellitus and hyperlipidemia were diagnosed according to relevant guidelines and consensus. Detection of biochemical indicators: Blood samples were collected from all patients on the next morning after fasting for at least 12 h. Glycosylated hemoglobin A1c (HbA1C), SUA, creatinine (CR), total bilirubin (TBIL), direct bilirubin (DBIL), indirect bilirubin (IBIL), triglyceride (TG), total cholesterol (TC), high density lipoprotein cholesterol (HDL-C) and low-density lipoprotein (LDL-C) were collected Cholesterol, lipoprotein (a), high-sensitivity C-reactive protein (hs-CRP) and cardiac ejection fraction.

### Assessment of coronary stenosis

Gensini score is a widely used angiographic scoring system to quantify the severity of CAD. It has been developed to characterize the complexity of CAD. It considers three main parameters for each coronary artery lesion: the severity score, the regional multiplication factor, and the collateral regulation factor [22–24]. The Gensini score was calculated by multiplying the location of the lesion with the degree of stenosis. The higher the Gensini score, the more severe the coronary artery lesion.

All patients underwent coronary angiography and signed the informed consent before operation. Biochemical, blood routine, urine routine, stool routine, coagulation index, echocardiography, X-ray, and other examinations were performed before operation. The operation was performed by qualified doctors in the catheterization room of the cardiology department of our hospital, and the procedure was carried out according to the interventional operation specifications. Then, according to the stenosis of the left main artery, anterior descending artery, circumflex artery, and right coronary artery (≥ 50%), single, double, and multiple artery lesions were diagnosed. According to the results of CAG, the degree of stenosis of each coronary artery was quantitatively evaluated. The degree of stenosis was defined as the most severe stenosis, with 1 point for stenosis diameter < 25%, 2 points for 25%≤ stenosis diameter < 50%, 4 points for 50%≤ stenosis diameter < 75%, 8 points for 75%≤ stenosis diameter < 90%, and 16 points for 90%≤ stenosis diameter < 99%. The stenosis diameter ≥ 99% was assigned 32 points. According to different coronary branches, the above scores were multiplied by the corresponding coefficients left main coronary artery lesion ×5; The proximal segment ×2.5, middle segment ×1.5, distal segment ×1 of left anterior descending artery. The first diagonal branch ×1, the second diagonal branch ×0.5; Proximal circumflex branch ×2.5, distal and posterior descending branch ×1, posterior collateral branch ×0.5; The proximal, middle, distal segments of the right coronary artery and the posterior descending artery were all ×1, and the sum of the scores of each diseased branch was the total score of coronary artery stenosis [22, 23]

### Statistical analysis

The measurement data in line with normal distribution were expressed as mean ± standard deviation (x ± s), and the comparison between groups was performed by t test. The measurement data in line with normal distribution were expressed as median (interquartile range) [M (Q25, Q75)]. The qualitative data such as coronary heart disease, hyperlipidemia, hypertension, diabetes, smoking history, and drinking history were expressed as rates. The chi-square test was used to compare the constituent ratios. Spearman correlation analysis was used for correlation analysis. Multivariate Logistic regression analysis was used to analyze the risk factors of coronary heart disease. SPSS 26.0(SPSS Inc., Chicago, Ill., USA) statistical software was used to analyze the data, the significance level was two-sided and set at *p*-values less than 0.05. Image created with GraphPad Prism (Version 10.0.0).

## Results

### Baseline characteristics of participants

After exclusion of ineligible patients, 860 patients were finally enrolled in this study. There were 625 (72.7%) cases in CHD group and 235 (27.3%) cases in non-CHD group. The baseline characteristics of the subjects were shown in Table 1. Compared with the control group, the coronary heart disease group had higher prevalence of smoking, diabetes, hypertension, hyperlipidemia and Gensini score (*P* < 0.05). The male patients, SUA, HbA1C, Gensini score, BMI, TG and hsCRP in CHD group were higher than those in control group (*P* < 0.05), while HDL-C was lower than that in control group (*P* < 0.05). There were no significant differences in age, heart rate, Cr, TC and LDL-C between the two groups (*P* > 0.05).

**Table 1:** Baseline Characteristics of study population

	Total population	CHD (n = 625)	Non-CHD (n = 235)	*P*-value
Age, years	64.0 ± 11.2	64.3 ± 12.0	63.0 ± 11.5	0.152
Male, n (%)	528(61.4)	444(51.6)	84(9.8)	< 0.001
Heart rate	79.9 ± 13.7	79.7 ± 14.0	80.3 ± 13.0	0.526
BMI (kg/m2)	25.5 ± 4.5	25.7 ± 4.4	25.0 ± 4.7	0.023
SBP (mmHg)	133.2 ± 21.1	134.1 ± 21.8	131.1 ± 19.0	0.049
DBP (mmHg)	78.5 ± 12.1	78.8 ± 12.2	77.7 ± 11.8	0.271
Previous History, n (%)				
Smoking	414(48.1)	357(42.5)	57(6.6)	< 0.001
Drinking	325(37.8)	274(31.9)	51(5.9)	< 0.001
Hypertension	536(62.3)	515(59.9)	121(2.4)	< 0.001
Diabetes	292(34.0)	239(27.8)	53(6.2)	< 0.001
UA (µmol/L)	343.1 ± 87.9	365.3 ± 84.0	284.1 ± 70.2	< 0.001
Male		376.40 ± 83.53	311.33 ± 77.29	< 0.001
Female		338.00 ± 77.14	268.97 ± 61.17	< 0.001
Gensini score	18(4.0,40)	30(14,48)	0(0,3)	< 0.001
HbA1C (%)	5.9(5.6,6.6)	5.9(5.7,6.7)	5.8(5.6,6.2)	< 0.001
TC (mmol/L)	4.2 ± 1.1	4.2 ± 1.1	4.3 ± 1.0	0.628
TG (mmol/L)	1.3(1.0,1.8)	1.32(1.0,1.9)	1.2(0.9,1.6)	< 0.001
HDL-C(mmol/L)	1.2 ± 0.3	1.1 ± 0.3	1.3 ± 0.4	< 0.001
LDL-C(mmol/L)	2.6 ± 1.0	2.6 ± 1.0	2.5 ± 0.9	0.668
Lp(a) (mmol/L)	105.5(50,260)	115(52,276)	90(45,216)	0.052
hsCRP(mmol/L)	1.4(0.6,4.8)	1.9(0.7,7.1)	0.8(0.4,1.8)	< 0.001
Cr(µmol/L)	76.0 ± 21.2	78.8 ± 22.0	68.7 ± 17.5	< 0.001
EF (%) M(IQR)	62(57,65)	61(55,65)	63(60,66)	< 0.001

Note: BMI: body mass index; SBP: systolic blood pressure; DBP: diastolic blood pressure; UA: uric acid; HbA1C: glycosylated hemoglobin; TC: total cholesterol; TG: triglyceride; LDL-C: low density lipoprotein cholesterol; HDL-C: high density lipoprotein cholesterol; DM: Diabetes mellitus; Lp(a): lipoprotein; Cr: creatinine; EF: ejection fraction of the heart. *P < 0.05, the differences were statistically significant

### Comparison of gensini scores grouped by quartiles of SUA

SUA was divided into group A, group B, group C and group D. The Gensini scores of the four groups were group D > group C > group B > group A, and the differences among the four groups were statistically significant (P < 0.05) (Table 2).

**Table 2:** Gensini scores of different SUA levels were compared [expressed as M (P25, P75)]

Group of groups		Gensini score (IQR)	Rank sum test	
			H	*P*
Male(n)				
	A (47)	14.00(8.00,42.00)	89.868	< 0.001
	B (93)	18.00(10.00,35.00)		
	C (141)	30.00(18.00,44.00)		
	D (163)	46.00(32.00,80.00)		
Female(n)				
	A (38)	10.00(6.00,20.00)	35.567	< 0.001
	B (62)	19.50(11.00,36.00)		
	C (44)	24.00(12.00,38.00)		
	D (37)	44.00(25.00,68.00)		

Note: IQR: Interquartile range; *P* < 0.05, the differences were statistically significant

The levels of serum uric acid and Gensini score in the diabetic group were higher than those in the non-diabetic group, there was statistical significance (P < 0.05) (Table 3).

**Table 3:** Comparison of uric acid and Gensini score between the two groups

Group of groups	Number of cases	UA (µmol/L, x ± s)	Gensini score
DM group	292	365.68	31.41
Non-diabetic group	568	331.49	25.37
t		5.22	2.85
*P*-Value		< 0.001	0.005

**Table 4:** Spearman correlation analysis of each risk factor with CHD and Gensini score

Characteristics	CHD		Gensini score	
	r	*P*	r	*P*
Gender	-3.323 ^a^	< 0.001^*^	-3.338 ^a^	< 0.001^*^
Age	0.060	0.081	0.043	0.207
Hypertension	0.137 ^a^	< 0.001	0.109	0.001
diabetes	0.148 ^a^	< 0.001	0.150 ^a^	< 0.001
Hyperlipidemia	0.110 ^a^	0.001	0.183 ^a^	< 0.001
Smoking	0.285 ^a^	< 0.001	0.285 ^a^	< 0.001
Drinking alcohol	0.203 ^a^	< 0.001	0.211 ^a^	< 0.001
UA	0.451^a^	< 0.001	0.583^a^	< 0.001
HbA1C	0.135 ^a^	< 0.001	0.146 ^a^	< 0.001
BMI	0.102 ^a^	< 0.001	0.130 ^a^	< 0.001
SBP	0.064	0.061	0.012	0.733
DBP	0.038	0.267	-0.009	0.798
TC	-0.030	0.380	0.031	0.369
TG	0.137 ^a^	< 0.001	0.209 ^a^	< 0.001
HDL-C	-0.245 ^a^	< 0.001	-0.344 ^a^	< 0.001
LDL-C	0.003	0.935	0.060	0.080
Lp(a)	0.066	0.052	0.105 ^a^	0.002
hsCRP	0.259 ^a^	< 0.001	0.358 ^a^	< 0.001

Note: Bivariate correlation analysis, ^a^*P*<0.01, *P: comparison with females

### Diagnostic value of SUA in coronary Heart Disease

**Fig. 1:**
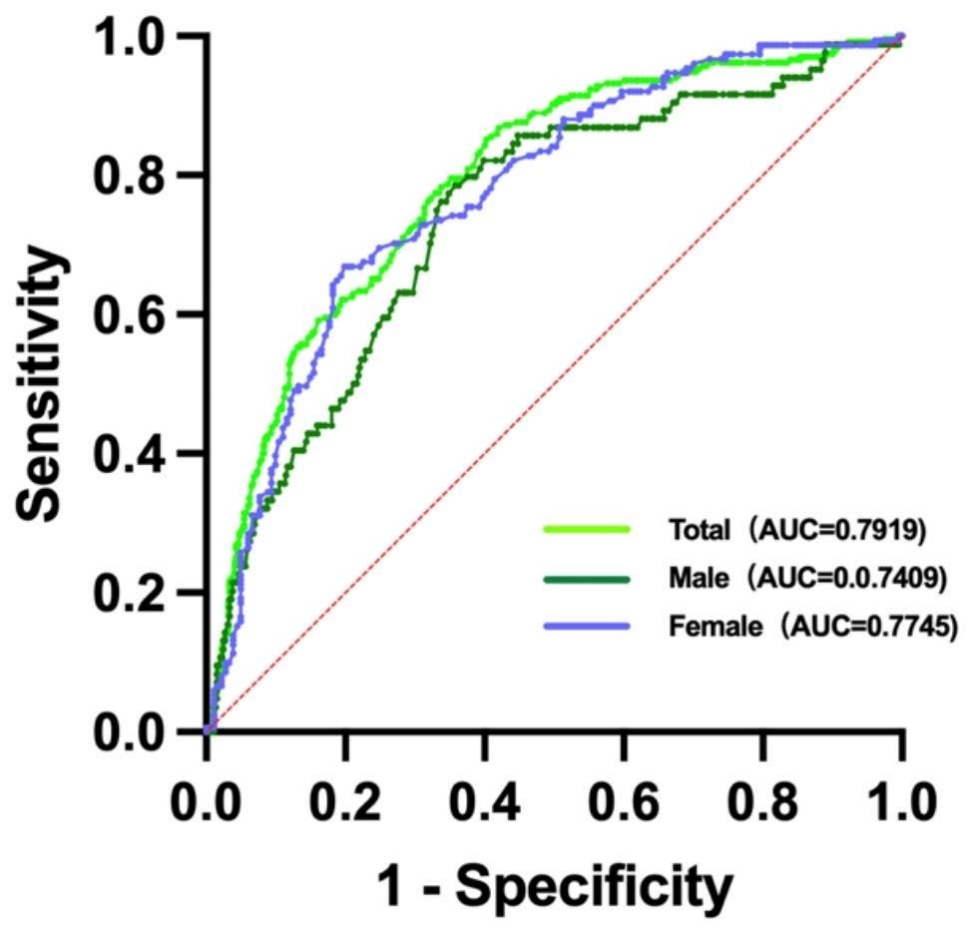
ROC curve of SUA for the diagnosis of CHD

The diagnostic value of SUA for coronary heart disease in the overall population, male population and female population was analyzed. Figure 1 show that, the area under the curve (AUC) and 95%CI were 0.792(0.758–0.825), 0.741(0.683–0.799) and 0.775(0.724–0.825), respectively. When the optimal cut-off values of SUA were 345.5µmol/L, 349.5µmol/L and 285.5µmol/L in the overall population, male population, and female population, respectively. The sensitivity of SUA in diagnosing coronary heart disease was 85.1%, 78.6%, 66.9%, and the specificity was 59.7%, 64.2%, 80.1%, respectively (Fig. 1).

### Spearman correlation analysis of each risk factor with CHD and Gensini score

Spearman correlation analysis showed that smoking, drinking, hypertension, diabetes, hyperlipidemia, BMI, TG, Lp (a), hsCRP, HbA1C, UA were positively correlated with CHD and Gensini score (*P* < 0.05). Gender and HDL-c were negatively correlated with CHD and Gensini score (*P* < 0.05). However, age, SBP, DBP, TC and LDL-C were not significantly correlated with CHD and Gensini score (*P* > 0.05) (Table 4).

Gender, age, smoking, SBP, GFR, BMI, UA, HbA1C, TG, LDL-C, Lp(a) and hsCRP were included to construct a multivariate linear regression equation. Results In the final model, gender, age, BMI, UA, LDL-C and hsCRP had statistically significant differences on Gensini score (P < 0.05) (Table 5).

**Table 5:** Multiple linear regression analysis of Gensini score and risk factors

Characteristics	β	SE	t	*P*-Value
Female*	-7.30	2.62	-2.79	< 0.001
Age	0.23	0.09	2.63	0.005
Smoking	1.53	2.48	0.62	0.009
UA	0.13	0.01	10.88	< 0.001
BMI	0.68	0.20	3.32	< 0.001
SBP	-0.002	0.04	-0.04	0.97
HbA1C	0.08	0.76	0.11	0.913
TG	1.32	0.75	1.77	0.077
LDL-C	1.91	0.92	2.08	0.038
Lp(a)	0.003	0.003	1.08	0.28
hsCRP	0.88	0.15	5.82	< 0.001
GFR	0.002	0.04	0.06	0.950

Note: * Male control

### Logistic regression analysis of risk factors associated with CHD

Gender, age, smoking, drinking, hypertension, diabetes, hyperlipidemia, BMI, TG, HDL- C, Lp (a), hsCRP, HbA1C, UA and other factors were included in the multivariate Logistic regression equation. The results showed that age, hypertension, hsCRP, SUA levels increased the risk of coronary heart disease. The difference was statistically significant (OR = 1.034,95%CI 1.016–1.052, *P* = 0.001; OR = 1.469,95%CI1.007-2.142, *P* = 0.046; OR = 1.064,95%CI1.026-1.105, *P* = 0.001; OR = 1.011,95%CI 1.008–1.014, *P* = 0.000). Compared with females, males had a significantly increased risk of coronary heart disease (OR = 0.5,95%CI 0.3-0.833, *P* = 0.008). Smoking increased the risk of coronary heart disease, and the difference was statistically significant (OR = 2.358,95%CI 1.282–4.339, *P* = 0.006) (Table 6).

**Table 6:** Logistic regression analysis of risk factors associated with CHD

Characteristics	β	SE	Wald	*P*-Value	OR	95%CI	
						Lower	Upper
Female	-0.692	0.26	7.082	0.008*	0.500	0.300	0.833
Age	0.033	0.009	13.469	0.001	1.034	1.016	1.052
Smoking	0.858	0.311	7.064	0.006	2.358	1.282	4.339
Drinking	-0.257	0.294	0.764	0.382	0.774	0.435	1.376
Hypertension	0.385	0.192	3.991	0.046	1.469	1.007	2.142
diabetes	0.062	0.284	0.048	0.827	1.064	0.610	1.857
Hyperlipidemia	0.239	0.221	1.165	0.280	1.270	0.823	1.959
BMI	0.026	0.021	1.735	0.241	1.025	0.983	1.069
UA	0.011	0.001	60.144	0.000	1.011	1.008	1.014
HbA1C	0.094	0.013	0.519	0.471	1.098	0.851	1.416
TG	0.050	0.100	0.248	0.619	1.051	0.864	1.278
HDL-C	-0.485	0.319	2.310	0.129	0.616	0.329	1.151
Lp(a)	0.000	0.000	1.034	0.309	1.000	1.000	1.001
hsCRP	0.062	0.019	10.925	0.001	1.064	1.026	1.105

Note: *P* < 0.05, the differences were statistically significant

## Discussion

The main findings of this study are as follows: (1) The proportion of male patients with coronary heart disease was higher than that of female patients, and the proportion of hypertension, diabetes, hyperlipidemia, smoking, and drinking was significantly higher than that of the control group (*P* < 0.01). (2) The levels of SUA and Gensini score in the CHD group were significantly higher than those in the control group (*P* < 0.05), and the level of SUA increased with the increase of the number of coronary artery lesions. Smoking, drinking, hypertension, diabetes, hyperlipidemia, BMI, TG, Lp (a), hsCRP, HbA1C, UA were positively correlated with CHD and Gensini score (*P* < 0.05). Gender and HDL-c were negatively correlated with CHD and Gensini score (*P* < 0.05), suggesting that the above indicators may be involved in the pathogenesis of coronary heart disease as risk factors. SUA level affects the severity of coronary artery disease, with the increase of SUA level, the severity of coronary artery disease increases. (4) The levels of uric acid and Gensini score in patients with multi-vessel coronary heart disease were significantly higher than those in patients with double-vessel and single-vessel coronary heart disease, and the serum uric acid level in patients with multi-vessel coronary heart disease was the highest. (5) Multivariate logistic regression analysis showed that when multiple risk factors were involved, the partial regression coefficients of hs-CRP, BMI, HDL-C and SUA were statistically significant. It is suggested that the above indicators are still closely related to the degree of coronary artery stenosis after comprehensive consideration of multiple factors.

Partial correlation analysis, single factor linear regression analysis and multiple stepwise regression analysis showed that serum uric acid level was an independent influencing factor of Gensini score, and serum uric acid level was closely related to Gensini score. Coronary artery disease in patients with high serum uric acid level was more severe, and the possibility of multi-vessel disease was higher. This result is similar to Ian J Neeland et al. [22]. Uric acid plays an important role in the pathophysiology of cardiovascular diseases and has been widely studied to be closely related to the occurrence and development of a variety of cardiovascular diseases. However, although this study and others have examined the relationship between uric acid and the Gensini score, a tool for assessing the severity of coronary heart disease, Alessandro Maloberti et al. [25]. failed to find a significant association. Although uric acid was significantly associated with CHD in this study, multiple studies have found that uric acid as a single risk factor has a relatively low OR value. It is suggested that as an independent cardiovascular risk factor, uric acid may play a small role in the occurrence of coronary heart disease. We suggest that elevated uric acid levels may be an indicator of cardiovascular risk, acting in conjunction with other risk factors. In addition, the results of this study may be confounded by other cardiovascular risk factors, such as hypertension, hyperlipidemia, diabetes mellitus, and the use of antihypertensive and lipid-lowering drugs. The combination of these factors has a higher predictive power for the risk of coronary heart disease.

Uric acid levels have been shown to be positively correlated with the severity of coronary artery disease [26–28]. Patients with higher levels of uric acid have higher Gensini scores and more diseased vessels, critical lesions, and total occlusive vessels. In our study, further breakdown of uric acid by gender confirmed this conclusion. The incidence of coronary heart disease in China is increasing day by day, and the age of onset of patients also shows a trend of younger [21]. With the gradual improvement of medical technology and the change of medical diagnosis and treatment ideas in China, the inpatient diagnosis, treatment, and treatment of coronary heart disease are gradually transformed into the pre-hospital prevention and treatment of coronary heart disease. Some studies have shown that uric acid, as the product of purine metabolism, is an independent biomarker capable of predicting morbidity and mortality in patients with various CVD [29]. The mechanism by which elevated uric acid causes and aggravates coronary heart disease is currently believed to be related to oxidative stress and inflammatory response caused by uric acid. Uric acid produces many oxygens free radicals through xanthine dehydrogenase and xanthine oxidase [30, 31], which participate in a series of inflammatory reactions and destroy the endothelium-mediated vasodilation function, leading to vascular endothelial dysfunction [20, 32, 33]. In addition, uric acid can also activate platelets, release a variety of cytokines, activate the coagulation system, and aggravate the adhesion and aggregation of platelets, thus increasing the risk of thrombosis and shedding, which is easy to cause coronary embolism and increase the incidence of cardiovascular events [34]. Uric acid can interfere with the synthesis of nitric oxide, inhibit the bioavailability of nitric oxide, activate the renin-angiotensin system, promote vascular smooth muscle cell proliferation and platelet aggregation, and eventually cause endothelial dysfunction [13, 35]. These pathways not only explain how hyperuricemia affects and promotes the occurrence and development of atherosclerosis, but also provide a rich theoretical basis for the predictive value of serum uric acid in the degree of coronary heart disease. In the URRAH [36] study, ROC curve analysis yielded a predictive value of SUA for Cardiovascular Mortality (CVM) of 5.8 mg/dL in patients with GFR higher than 60 ml/minute. High SUA levels are a risk factor for cardiovascular and all-cause mortality in patients at cardiovascular risk, which is similar to the finding in this study that uric acid is at the predicted cutoff for coronary heart disease, more pronounced in female patients. Although CKD is a major determinant of the presence and extent of hyperuricemia, they both contribute to excess CV morbidity and mortality.

This study is a cross-sectional survey of suspected coronary heart disease (CHD) population at high risk of coronary angiography. Coronary heart disease was accurately judged by the gold standard of coronary angiography and the severity of coronary artery disease was evaluated by Gensini score, which is the latest data to objectively reflect the correlation between serum uric acid level and CHD and the severity of coronary artery disease in western China. But there are potential limitations: 1. This study was limited by its single-center and retrospective nature, and all patient information came from the electronic medical record system of the tertiary hospitals. The sample size of the study was still relatively small, and the lack of randomness was prone to bias. (2) In this study, there was no control of diet and some potential confounding factors before blood collection, which may affect UA metabolism due to purine intake, and the serum uric acid results did not reach two measurements in different days; (3) The control group of this study was the patients whose coronary angiography results did not meet the standard of coronary heart disease, and they were not completely healthy people. Moreover, the multiple metabolic abnormalities of patients with coronary heart disease may confound the analysis results, which may lead to inevitable statistical errors. (4) Some studies have reported that uric acid is associated with strong inflammation (white blood cells, eosinophils, CRP) and the severity of coronary artery involvement. Juan Chen et al. [37]. showed that neutrophil-lymphocyte ratio (NLR) was an independent predictor of coronary heart disease (OR = 1.18, 95%CI: 1.09–1.27, p = 0.009) and high GS (OR = 1.10, 95%CI: 1.01–1.16, p = 0.032). In addition, GS was slightly positively correlated with NLR, white blood cell (WBC) count, and the proportion of neutrophils and monocytes. In addition, Suhas Hardas [38] et al. showed that Echocardiographic non-coronary calcium score (ECS) was significantly positively correlated with Gensini score. Gensini scores increased as ECS increased. Some inflammatory variables and ECS were not included in this study, which is a defect of this study. In the future direction of blood uric acid research, we should consider increasing the laboratory detection of ECS, homocysteine, insulin level, CRP, eosinophils, blood coagulation and fibrinolysis system related factors and a series of biological markers related to inflammation, oxidative stress and insulin resistance. And a larger sample size to further explore the independent factors associated with the progression of coronary artery disease.

It has been suggested that reducing uric acid levels may be beneficial in the prevention of cardiovascular events, and uric acid-lowering treatment can reduce the incidence of coronary heart disease and its effect on mortality [39]. However, the ALL-HEART [40] large, randomized trial, which compared cardiovascular outcomes in patients with ischemic heart disease without a history of gout who received purinol versus usual care, showed that the primary outcome of nonfatal myocardial infarction, stroke, or cardiovascular death did not differ significantly between participants randomized to isopurinol and those randomized to usual care. Second, the results of this study do not support the trial of isopurinol for the prevention of adverse cardiovascular consequences in ischemic heart disease patients with asymptomatic hyperuricemia (gout). Although the ALL-HEART trial may not have met expectations, it will contribute to our understanding of the association between hyperuricemia and cardiovascular disease and to the development of urate-lowering preventive approaches and therapeutic strategies to reduce the risk of these patients. Finally, there is currently no strong evidence of a modest benefit of medical treatment of hyperuricemia in reducing CV morbidity and mortality [17]. In conclusion, more studies should be conducted to clarify the involvement of this molecule in the CV disease spectrum and its possible role as a target for CV prevention strategies.

In summary, this study provides new insights into the correlation between uric acid and Gensini score and its gender differences. Uric acid, as a biomarker, may have potential clinical value in cardiovascular risk assessment. Meanwhile, studies on gender differences are of great significance for further understanding the relationship between uric acid and coronary artery disease and the gender specificity of cardiovascular disease. Future studies need to further explore the predictive value of uric acid and consider gender factors more comprehensively to provide more accurate basis for individualized cardiovascular risk assessment and intervention.

## Conclusion

In patients with coronary heart disease, SUA is positively correlated with Gensini score. SUA is an independent influencing factor of Gensini score for coronary artery stenosis and can predict the severity of coronary artery disease.

## Data Availability

The database used and analyzed in this study is not publicly available, as its information may prejudice the privacy of participants and their consent to participate in the study. However, the data are available from the corresponding author upon request.

## Abbreviations

CHD: Coronary heart disease
SUA: Serum uric acid
BMI: Body mass index
SBP: Systolic blood pressure
DBP: Diastolic blood pressure
HbA1C: Glycosylated hemoglobin
TC: Total cholesterol
TG: Triglyceride
LDL-C: Low density lipoprotein cholesterol
HDL-C: High density lipoprotein cholesterol
Lp(a): Lipoprotein
Cr: Creatinine
EF: Ejection fraction of the heart
I: Confidence interval
IQR: Interquartile range
OR: Odds ratio
